# Supplementary material for: Cholesterol-dependent enrichment of understudied erythrocytic stages of human Plasmodium parasites
Source: Sci Rep. 2020 Mar 12;10:4591. doi: 10.1038/s41598-020-61392-6 (PMC7067793; doi:10.1038/s41598-020-61392-6)

Cholesterol-dependent enrichment of understudied erythrocytic stages of human *Plasmodium* parasites

Audrey C. Brown[1], Christopher C. Moore[2], Jennifer L. Guler*[1,2]
[1]Department of Biology, University of Virginia, Charlottesville, VA.
[2]Division of Infectious Diseases and International Health, University of Virginia, Charlottesville, VA

Supplemental Materials

Supplementary Materials not included in this document:

Supplementary Method 1. Detailed SLOPE Protocol (separate .docx attachment)

Supplementary Data 1. Complete p180 Metabolite Data (separate .csv attachment)

All other Supplementary Materials are included below:

Supplementary Table 1. 10 most influential metabolites for principal components 1 and 2

PC1	PC2
PC.aa.C34.1	*C18*
PC.aa.C38.5	*C18.2*
PC.aa.C38.6	*C18.1*
PC.aa.C32.2	*C16.1.OH*
PC.aa.C36.5	*SM.C16.1*
PC.aa.C30.0	*lysoPC.a.C28.1*
PC.aa.C32.1	*C16*
PC.aa.C34.2	*SM.C20.2*
PC.aa.C36.3	*PC.aa.C28.1*
PC.ae.C38.0	*lysoPC.a.C24.0*

Supplementary Table 2. Quantification of cholesterol from human plasma and AlbuMAX II LipidRich BSA

Source	Average	S.E.M.	*N*
Human Plasma	3300 µM	650 µM	4
AlbuMAX II	13.5 µM	1.43 µM	4

Supplementary Figure 1. SLOPE enrichment is effective irrespective of parasite line. SLOPE enrichment performed on three different ring stage synchronized *P. falciparum* lines grown in the same blood and AlbuMAX II media batches. For each graph: *N*=3. Error bars represent S.E.M.

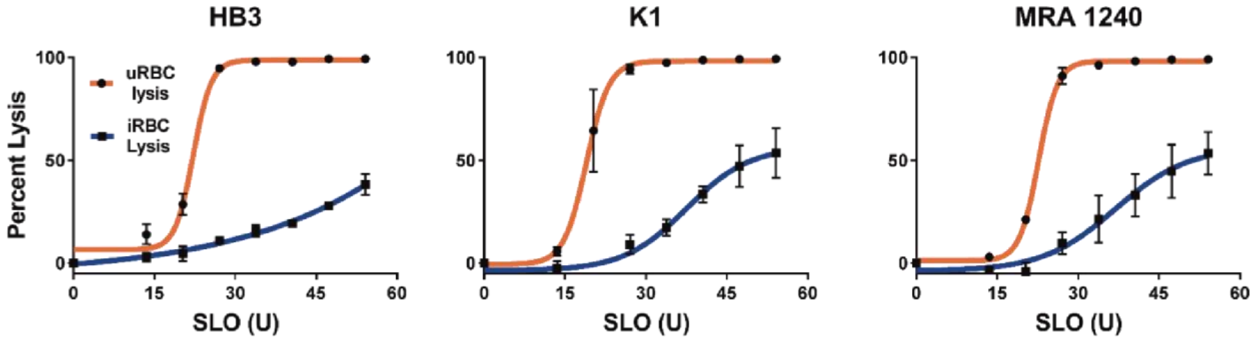

Supplementary Figure 2. SLOPE enrichment shows no bias across stages of the intraerythrocytic cycle. *P. falciparum* line MRA 1240 cultures either (A) synchronized by one sorbitol treatment or (B) left asynchronous were staged by microscopy before and after SLOPE enrichment. For each graph: *N*=3 (200 parasites counted per replicate). Error bars represent S.E.M.

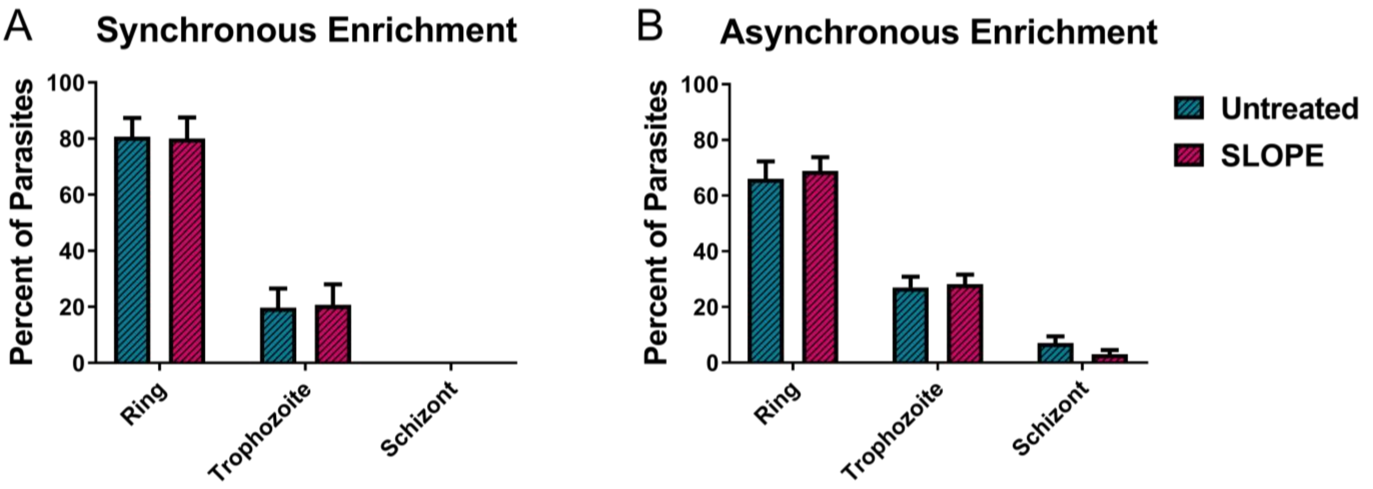

Supplementary Figure 3. SLOPE enrichment does not affect growth or staging over multiple replication cycles. (A) Three days of *P. knowlesi* line yH1 parasite growth from untreated controls or SLOPE enriched samples diluted with uninfected erythrocytes (*N* = 3, error bars represent S.E.M.). (B) Stage distribution for asynchronous Untreated and SLOPE enriched *P. knowlesi* line yH1 every 24h during 3 days of re-culture growth. (*N* = 3, error bars represent S.E.M.). R = ring; T = trophozoite; S = schizont. (C) The percentage of parasites in the ring stage for synchronized Untreated and SLOPE enriched *P. falciparum* line MRA 1240 every 48h during 6 days of re-culture growth. (*N* = 3, error bars represent S.E.M.).

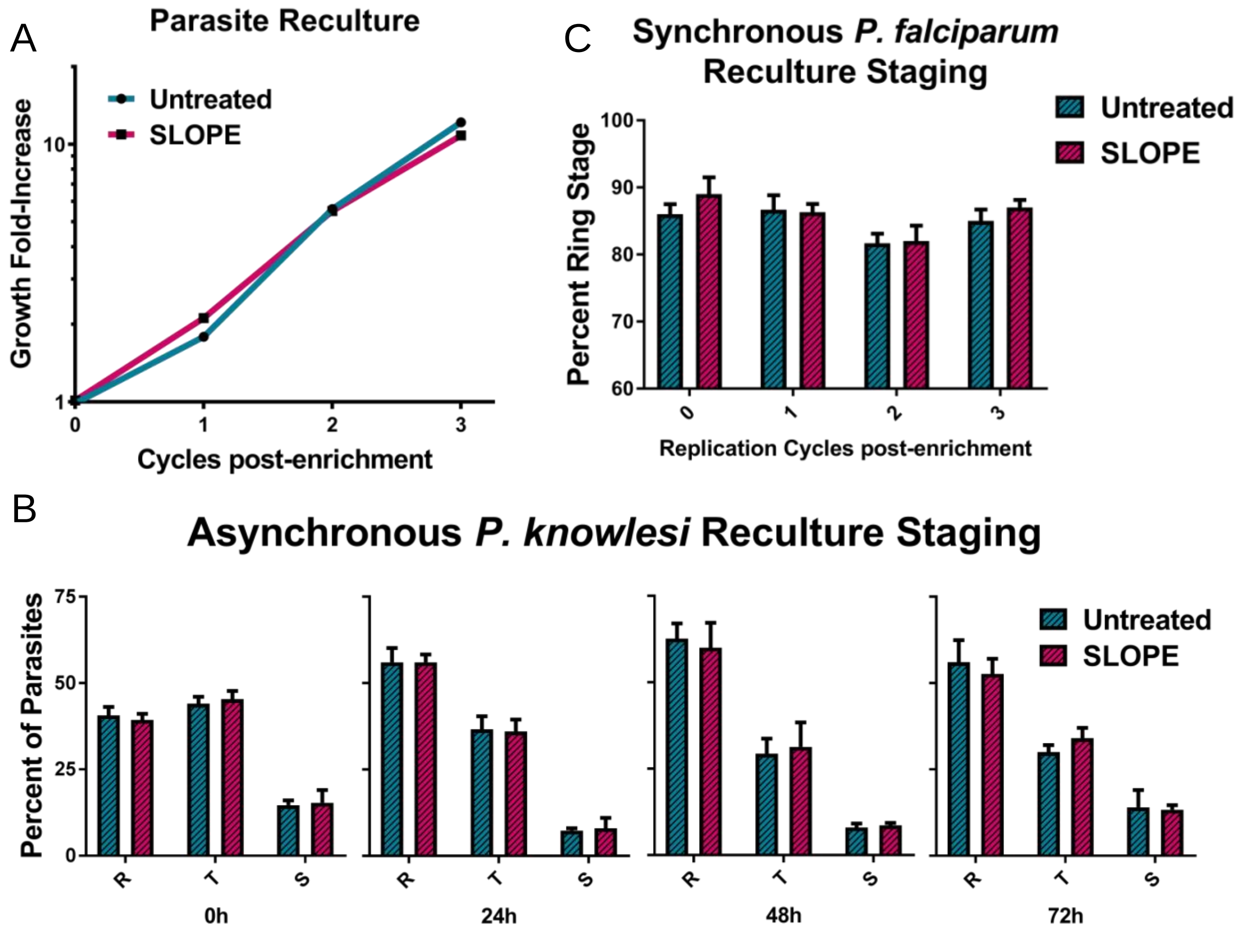

Supplementary Figure 4. SLOPE enrichment is effective irrespective of parasite line. SLOPE enrichment performed on clinical erythrocytes incubated in cRPMI for differing lengths of time. For infected erythrocytes (iRBCs), each line represents one trial; for uninfected erythrocytes (uRBCs), lysis curves from all time points were combined as no change was observed over time: *N*=4. Error bars represent SEM.

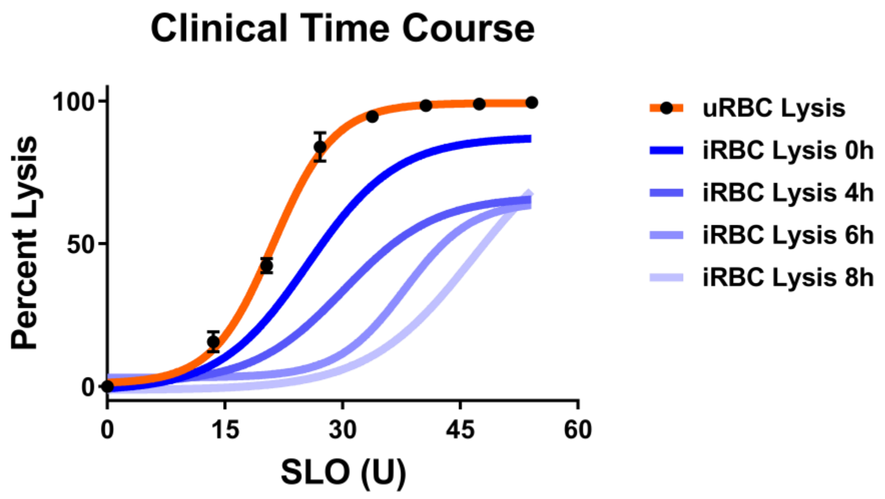

Supplement: Supplementary file 3 — Supplementary Figures and Tables [file 41598_2020_61392_MOESM3_ESM.pdf]
